# Factors Influencing Sulforaphane Content in Broccoli Sprouts and Subsequent Sulforaphane Extraction

**DOI:** 10.3390/foods10081927

**Published:** 2021-08-19

**Authors:** Jan Tříska, Josef Balík, Milan Houška, Pavla Novotná, Martin Magner, Naděžda Vrchotová, Pavel Híc, Ladislav Jílek, Kateřina Thorová, Petr Šnurkovič, Ivo Soural

**Affiliations:** 1 Global Change Research Institute CAS, 603 00 Brno, Czech Republic; triska.j@czechglobe.cz (J.T.); vrchotova.n@czechglobe.cz (N.V.); 2 Faculty of Horticulture, Mendel University in Brno, 691 44 Lednice, Czech Republic; josef.balik@mendelu.cz (J.B.); pavel.hic@mendelu.cz (P.H.); petr.snurkovic@mendelu.cz (P.Š.); 3 Food Research Institute Prague (FRIP), 102 00 Prague 10, Czech Republic; milan.houska@vupp.cz (M.H.); Pavla.Novotna@vupp.cz (P.N.); 4 Department of Pediatrics, University Thomayer Hospital, First Faculty of Medicine, Charles University, 140 59 Prague 4, Czech Republic; martin.magner@ftn.cz or; 5 Department of Pediatrics and Inherited Metabolic Disorders, First Faculty of Medicine, Charles University, General University Hospital, 128 08 Prague 2, Czech Republic; 6 Pure Food Norway, 1400 Ski, Norway; ladislav.Jilek@purefoodnorway.no; 7 National Institute for Autism, 182 00 Prague 8, Czech Republic; katerina.thorova@nautis.cz

**Keywords:** broccoli and radish sprouts, sulforaphane, myrosinase, extraction, epithiospecifier protein

## Abstract

Broccoli sprouts contain 10–100 times higher levels of sulforaphane than mature plants, something that has been well known since 1997. Sulforaphane has a whole range of unique biological properties, and it is especially an inducer of phase 2 detoxication enzymes. Therefore, its use has been intensively studied in the field of health and nutrition. The formation of sulforaphane is controlled by the epithiospecifier protein, a myrosinase co-factor, which is temperature-specific. This paper studies the influence of temperature, heating time, the addition of myrosinase in the form of *Raphanus sativus* sprouts in constant ratio to broccoli sprouts, and other technological steps on the final sulforaphane content in broccoli sprout homogenates. These technological steps are very important for preserving sulforaphane in broccoli sprouts, but there are some limitations concerning the amount of sulforaphane. We focused, therefore, on the extraction process, using suitable β-cyclodextrin, hexane and ethanol, with the goal of increasing the amount of sulforaphane in the final extract, thus stabilizing it and reducing the required amount sulforaphane needed, e.g., as a dietary supplement.

## 1. Introduction

By monitoring quinone reductase induction in cultured murine hepatoma cells in biological assays, Zhang et al. [1] were able to isolate sulforaphane (SR), a significant and potent phase II enzyme inducer, from broccoli. Approximately 9 mg of SR was isolated from 640 g of fresh broccoli florets (ca. 14 μg/g of fresh weight (f.w.)). Later, Fahey et al. [2] found that three-day-old broccoli sprouts (cultivar Saga) had an inducer content ca. 15 times higher than adult plants. Significant differences in the SR content of both broccoli and broccoli sprouts have been published in the literature; these differences can be explained in many ways, including the method of sample preparation. For example, Chiang et al. [3] either homogenized one gram of freshly harvested broccoli in 10 mL of warm (50 °C) distilled water for 5 min using a mixer or they used frozen broccoli, to which they added 25 units of thioglucosidase before homogenization (to ensure complete hydrolysis). They found the SR content of broccoli ranged from 1.89–3.7 mg/g, with the highest being the Super Dome variety, which was more than two orders of magnitude higher than the values initially stated by Zhang et al. [1]. This shows that sample preparation plays a very important role, which is often overlooked.

The glucosinolates (GL) content and its transformation, especially the transformation of glucoraphanine (glucoraphanin) (GR) and, therefore, the final SR content in broccoli sprouts, is affected by various factors, including pre-harvest factors, which can be very important. Although plant species and genotype significantly affect GR content and distribution, the effects of pre-harvest factors, including light, temperature, nutrition (i.e., sulfur (S) and nitrogen (N)), water, and phenological stage, cannot be ignored. There is limited literature regarding the effect of pre-harvest conditions on broccoli sprouts. It is quite clear that light is needed to achieve higher GR contents in broccoli sprouts. Pérez-Balibrea et al. [4] reported 33% higher concentrations of total GL relative to broccoli sprouts grown in the dark. 

GR is a sulfur-rich compound found in *Brassicaceae* plants; thus, its accumulation depends largely on the sulfur status of the whole plant and the N:S ratio. Schonhof et al. [5] found that an N:S ratio between 7:1 and 10:1 could promote plant yield and enhance GL accumulation in broccoli. Foliar application of a 5 mM and 10 mM methionine (a sulfur-containing amino acid) solution resulted in increased GL concentrations in 7-day-old broccoli sprouts, obtaining significantly higher amounts of GL (by 23% and 21%, respectively) than the non-treated sprouts on the same sampling date (Pérez-Balibrea et al. [6]). The same authors also showed that broccoli sprouts treated the standard elicitors, such as salicylic acid, methyl jasmonate, and chitosan, were highly effective, since every dose, on each sampling day, gave rise to a higher total GL content than that obtained in the control sprouts. Natella et al. [7] extended the study of the abovementioned elicitors to include sucrose, mannitol, and 1-aminocyclopropane-1-carboxylic acid. They identified sucrose as the most potent elicitor, which induced a significant increase in total and specific GL, in contrary to other studied elicitors.

Guo et al. [8] evaluated GL metabolism in broccoli sprouts under NaCl treatment and found the highest SR content (14 mg/g f. w. against control—10 mg/g f.w.) in 3-day-old broccoli sprouts using 100 mmol/L of sodium chloride solution, which decreased with the age of the broccoli sprouts. When the broccoli seeds were soaked in slightly acidic electrolyzed water (SAEW) with chlorine concentrations of 50.33 mg/L at a pH = 5.52, for 3 h at 25 °C, relative humidity of 80%, and in the dark, the final SR concentration increased to 11.49 mg/g d.w. (a 61.2% increase compared to controls) [9]. The authors harvested broccoli sprouts with roots after eight days, stored them at −80 °C, and then freeze-dried them before analysis. The issue of global changes is also reflected in studies examining the ability to influence sulforaphane content in broccoli sprouts. Almuhayawia et al. [10] grew three varieties of broccoli sprouts (Southern Star, Prominence, and Monotop) in a growth chamber at 25 °C, under a 16 h light/8 h light cycle, relative humidity of 60%, ambient carbon dioxide concentration of 400 ± 27 μmol CO_2_ mol^−1^, and elevated concentration carbon dioxide levels of 620 ± 42 μmol CO_2_ mol^−1^ air for nine days. The Monotop variety yielded an approximately threefold increase in sulforaphane (approx. 6 μmol/g f.w.) with half the content of sulforaphane nitrile (ca. 1.8 μmol/g f.w.), and with increased myrosinase activity and decrease in ESP activity.

Glucosinolates formed and accumulated in broccoli sprouts, especially GR, must undergo a somewhat complicated enzymolysis that results in isothiocyanates, especially SR. To achieve an optimal yield of SR, we would have to know the exact content of its precursor, GR, the amount and activity of myrosinase, the amount and activity of epithiospecifier protein (ESP), as well as the ratio of sprout biomass to water, the pH of the reaction mixture, the temperature, etc.

Several authors have conducted studies on the optimal enzymolysis conditions for sulforaphane production recently, e.g., Hanschen et al. [11] and Tian et al. [12]. Tian et al. [12] found that the maximum sulforaphane content (246.95 µg/g d.w.) was reached using a solid–liquid ratio of 1:30, a hydrolysis time of 1.5 h, an ascorbic acid content of 3.95 mg/g d.w., and a temperature of 65 °C. The highest content of SR in broccoli sprouts was 233.80 µg/g d.w. in 5-day-old sprouts and 1555.95 µg/g d.w. after four days of storage. During the preparation of broccoli sprout juice, Bello et al. [13] found that the yield of SR and sulforaphane nitrile (SRN) was only approximately 25% calculated based on the amount of GR in the original material. The loss during juice preparation was explained as (1) spontaneous conversion to sulforaphane-amine or (2) conjugation of the glutathione and proteins naturally present in the juice. The total amount of SRN extracted in the juice from 100 g of fresh sprouts was about 7 ± 3 μmol (0.12 ± 0.04 μmol/mL juice). After the addition of radish, rocket, and rape sprouts to the broccoli sprouts during GR hydrolysis, the yield of sulforaphane was increased by approximately two times compared to broccoli sprouts alone; the addition of mustard sprouts did not improve sulforaphane levels [14].

The formation of SR is controlled by ESP, which is present in some cruciferous plants, including broccoli, which is temperature-specific and converts the intermediate of glucoraphanin hydrolysis to a sulforaphane nitrile at the expense of SR.

Matusheski et al. [15] studied the effect of temperature on the formation of SR and sulforaphane nitrile in fresh broccoli florets and broccoli sprouts from several commercial cultivars. They found that heating fresh broccoli florets or broccoli sprouts to 60 °C prior to homogenization increased the content of SR and decreased the content of sulforaphane nitrile, while heating to 70 °C and above decreased the formation of both compounds in broccoli florets, but not in broccoli sprouts. ESP plays an important role in the “in vitro” study of GR to SR conversion, but its importance appears to decline in in vivo studies, e.g., SR bioavailability studies. 

The work of Fahey et al. [16], which is one of the most important in this area, does not mention ESP at all. The authors experimented with both SR- and GR-enriched broccoli sprout extracts, broccoli seed extracts, and freeze-dried broccoli sprouts as supplements. The highest bioavailability of SR (about 40% on molar basis) was found after 10 min in pre-hydrolyzed freeze-dried broccoli sprouts, mixed with pineapple-lime juice containing vitamin C.

It is clear from the above introduction that there is still no uniform nor universal procedure for the preparation of broccoli sprouts into usable food supplements guaranteeing high SR content. The lack of unified procedures has many factors, many of which are easily managed at laboratory production levels but difficult to implement on a commercial scale. Leaving aside the variety of broccoli sprouts, processing temperatures and hydrolysis of GR play a crucial role. The main goal of our work was to study, the effect of temperature on SR content in the final lyophilized product and extracts. The next goal was trying move production from a laboratory scale into a semi pilot plant mode and prepare a product with content of SR, which is in microbial limits and described basic sensory information about application.

## 2. Material and methods

### 2.1. Preparation of Powder Mixtures Containing Sulforaphane

#### 2.1.1. Original Procedure (OP)

A mixture of 450 g of lyophilized broccoli sprouts (Synergized Ingredients, Moab, UT, USA) and 50 g of white radish sprouts (Spirer AS, Gardvik, Norway) (9:1) was exposed to a temperature of 60 °C in an oven (MLW WS100, Dresden, Germany) for 18 min. The temperature inside the powder during that time reached more than 59 °C (time at 60 °C was 20 min). After cooling to 30 °C, the powder was mixed with water from the tap (1:10) at 20 °C for 5 min. The temperature was continuously measured during this time and ranged from 21.1 to 22.1 °C. The resulting suspension was deep-frozen (−20 °C) and then lyophilized (Sprout mixtures lyophilization was done in equipment produced by Kohout Company Ltd. (Prague, Czech Republic) type PD with chamber volume 0.93 m^3^ and total shelfs surface 5.86 m^2^, with a power input of 2.5 kW, for 3 days) to give about 500 g of a dry mixture, which was then analyzed.

#### 2.1.2. Fresh Sprouts Procedure (FSP)

A mixture of 9 kg of fresh broccoli sprouts and 1 kg of white radish sprouts (9:1) were stored at 5 °C with 25 kg of Ca(ClO)_2_ solution (150 mg/liter, pH 8.76, 19.5 °C) for 2 min; then, the sprouts were washed with water from the tap to a pH = 6.8. The sprouts were heated using a water bath to a temperature of about 60 °C for 10 min, then drained of excess water. The temperature was measured five times during this 10 min and ranged from 60.1 to 61.1 °C. Subsequently, the sprouts were pressed, and the resulting juice with a total volume of 6 L; 20.43 g of β-cyclodextrin (final concentration 3 mM) was added. Extraction with β-cyclodextrin was performed for 1 h with stirring. Subsequently, the juice was deep-frozen (−20 °C) and then lyophilized (Sprout mixtures lyophilization was done in equipment produced by Kohout Company Ltd. (Prague, Czech Republic) type PD with chamber volume 0.93 m^3^ and total shelfs surface 5.86 m^2^, with a power input of 2.5 kW, for 3 days) to about 220 g of a dry mixture, which was subsequently analyzed. The purities of the reagents were as follows: Ca(ClO)_2_ (70%) Fichema Ltd., Brno, Czech Republic; β-cyclodextrin (p.a., Sigma-Aldrich Ltd., Prague, Czech Republic).

#### 2.1.3. Original Procedure at a Higher Temperature (OPHT) 

A mixture of 450 g of lyophilized broccoli sprouts and 50 g of white radish sprouts (9:1) was exposed to 100 °C in an oven (MLW WS100, Dresden, Germany) for 60 min. The temperature inside the powder reached more than 99 °C (time at the endurance at 100 °C was 40 min). After cooling to 30 °C, the powder was mixed with water from the tap (1:10) at 50 °C. The mixture was heated to 100 °C, for 10 min, then cooled below 30 °C. The resulting suspension was deep-frozen (−20 °C), then lyophilized (Sprout mixtures lyophilization was done in equipment produced by Kohout Company Ltd., Prague, Czech Republic) type PD with chamber volume 0.93 m^3^ and total shelfs surface 5.86 m^2^, with a power input of 2.5 kW, for 3 days) to give about 500 g of a dry mixture, which was subsequently analyzed. Two temperature ranges were monitored: dry sprouts temperature and suspension temperature. The temperature of the dry lyophilized sprouts was continuously measured, reaching a temperature of 99.1 °C in 51 min. and increasing up to 102.5 °C in 111 min (time at 100 °C was 60 min). The temperature was then decreased to 40.2 °C over the next 40 min. The temperature of the suspension was continuously measured, reaching 100 °C in 15 min, and remained constant for up to 25 min (time at 100 °C was 10 min), then gradually decreased to 29 °C over the next 27 min.

#### 2.1.4. Modified Original Procedure (MOP)

This modified procedure at 60 °C was also used at the Faculty of Horticulture Lednice, Mendel University in Brno (note: this procedure was not to intended to optimize SR content in the mixture, but as a preparation for the extraction procedures using β-cyclodextrin and solvents (ethanol and hexane)). In contrast to drying at 60 °C, which took place on two baking trays covered with aluminum foil (the thickness of the plant material on the tray was up to 3 cm), the modified process (MOP) used one container, where the thickness of the plant material in the container was about 12 cm. The temperature of the powder was not continuously monitored, and therefore, the drying took place in a preheated oven (Memmert UFE 400, Memmert Ltd., Schwabach, Germany) for 30 min. After drying, the temperature in the middle of the batch (container) was measured to be less than 40 °C.

#### 2.1.5. Original Procedure at Higher Temperature in Semi Pilot Plant Mode (OPHT—Pilot Mode) 

The OPHT procedure was adapted to semi-pilot plant mode. A mixture of 6.25 kg of lyophilized broccoli sprouts and 0.694 kg of radish sprouts (approx. 9:1) was exposed to a temperature of 100 °C in a dry heat treatment device produced by FRIP, Czech Republic. The temperature inside the mixture reached 100 °C after about 50 min, the holding time at 100 °C was 40 min. After cooling, 4.1 kg of the powder was then added to 40.9 L of water from the tap (1:10) at 41.9 °C. This produced 45 kg of suspension, which was heated to 100 °C for 10 min in an aseptic cooking mixer (type AV-50; FRIP, Prague, Czech Republic). During heating, a certain amount of the suspension was lost through the open part of the device (to reduce the risk of overheating and overpressure); the final weight of the suspension was 28.25 kg. After cooling the suspension to 20 °C, the resulting suspension was deep-frozen (−20 °C) and lyophilized (Sprout mixtures lyophilization was done in equipment produced by Kohout Company Ltd. (Prague, Czech Republic) type PD with chamber volume 0.93 m^3^ and total shelfs surface 5.86 m^2^, with a power input of 2.5 kW, for 3 days) to give about 2.7 kg of the dry mixture, which was then analyzed.

### 2.2. Extraction of Sulforaphane from the Lyophilized Mixture

The produced powder mixture of broccoli and radish sprouts, processed as described in the OP, was used as the starting material for the extraction of SR using various solvents (ethanol and hexane) and cyclodextrin at room temperature.

#### 2.2.1. Hexane

The original lyophilized mixture was added to water to produce 1.8 L of the suspension, to which 700 mL of hexane (99%, n-hexane for HPLC, VWR) was then added. Extraction with hexane was performed on a shaker for 1.5 h; the resulting mixture was centrifuged (3500 rpm, 5 min), and the upper hexane (oil) layer was separated (420 mL). Hexane was gradually removed using vacuum evaporation with continuous addition of ethanol (>99.7%, absolute, for HPLC, VWR), and the newly formed ethanol extract (total volume 250 mL) was subsequently analyzed.

#### 2.2.2. Cyclodextrin

The original lyophilized mixture was added to water to produce 1.5 L of the suspension, to which β-cyclodextrin (final concentration 3 mM) was added (p.a., Sigma-Aldrich). Extraction by β-cyclodextrin was performed on a shaker for 1.5 h; the resulting mixture was centrifuged (3500 rpm, 5 min), the aqueous portion was decanted (700 mL), frozen and lyophilized, and then the resulting lyophilized was analyzed.

#### 2.2.3. Ethanol

The suspension (1.8 L) formed by adding water to the original lyophilized mixture was frozen and then lyophilized. To a portion of the newly formed lyophilizate (25.07 g), 300 mL of ethanol (>99.7%, absolute, for HPLC, VWR) was added, and the extraction was performed on a shaker for 24 h. The resulting suspension was centrifuged (3500 rpm, 5 min), and the ethanol supernatant was separated and subsequently analyzed.

### 2.3. Preparation of Samples for HPLC Analysis

From the resulting mixtures, SR was first extracted with dichloromethane, then concentrated on solid-phase extraction (SPE) columns (elution with methanol), and then measured using HPLC.

To 0.2 g of sample, 2 mL of distilled water was added and let stand for 15 min; then, 5 mL of dichloromethane (DCM) was added with shaking. After 15 min, centrifugation was performed, and 5 mL of DCM (for HPLC, Merck) was added to the aqueous portion again (this step was repeated four times); as such, the DCM extraction was a five-step extraction to achieve optimal recovery of SR into DCM). The individual DCM fractions were combined and dried over anhydrous sodium sulfate (p.a., Sigma-Aldrich).

To compare the extraction procedures in the analysis, two modifications were tested (M1 and M2). In the first modification, the addition of water (M1) was omitted (procedure designated as Sample No. 1), and in the second sample, water was added to the mixture after the first extraction with 5 mL of DCM (M2) (procedure designated as Sample No. 2). 

After activating the SPE columns (Supelco Discovery DSC-Si, 500 mg) with 10 mL of DCM, the DCM extract of sulforaphane was applied, then the column was washed with several mL of pure DCM then with ethyl acetate (for HPLC, Merck) until a colorless ethyl acetate solution was flowing out. Subsequently, the SPE columns were dried, followed by elution of SR three times with methanol (for HPLC, Merck) 1.5 mL, 1.0 mL, and 0.5 mL. The volume of the eluate was recorded.

### 2.4. HPLC Analysis of Sulforaphane and Sulforaphene

The samples were analyzed using an HP 1050 HPLC (Ti-series) (Hewlett-Packard, Palo Alto, CA, USA), diode array detector Agilent 1100 series on a Luna C18(2) column, 150 mm × 2 mm, 3 μm (Phenomenex, Torrance, CA, USA), and a water–acetonitrile–*o*-phosphoric acid mobile phase. Mobile phase A used 5% acetonitrile + 0.1% of *o*-phosphoric acid; mobile phase B used 80% acetonitrile + 0.1% of *o*-phosphoric acid (in volume%). The gradient was increased from 0% of B to 45% of B over 30 min. The flow rate was 0.250 mL/min, and the column temperature was 25 °C. The injection volume was 5 μL, the detection wavelengths were set at 245 nm, and the scanning range was 190–600 nm. The purities of the reagents were as follows: acetonitrile (for HPLC, Merck); *o*-phosphoric acid (for HPLC, Fluka). The analytical process was validated and the main parameters are the following: R^2^ = 0.9984; LOD = 1.413 μg/mL; LOQ = 4.709 μg/mL.

### 2.5. Statistical Analysis

Each technological operation (preparing mixture, extraction, etc.) was done twice. All SR analyses were done in triplicate, and results were expressed as means and standard deviations. The significance of differences was tested using Student’s *t*-test with a level of significance of *p* ˂ 0.05; Microsoft Excel was used for data processing.

### 2.6. Sensory Initial Testing

The prepared lyophilized broccoli sprouts were sensory tested by ten volunteer assessors who evaluated them as possible food additives for their future usage in the different kinds of food. Their observation and knowledge were described in results and discussion.

### 2.7. Microbial Quality Evaluation

The total number of microorganisms was determined by culturing on Plate count agar (HiMedia), potting 30%, culturing for 2–3 days. Fungi and yeast were cultured on glycerol agar DG 18 (Dichloran Glycerol Chloramphenicol, Merck), 0.1 mL per already poured dish. After culturing for 4 to 5 days, the number of colonies was read.

## 3. Results and Discussion 

### 3.1. Stability of Sulforaphane in the Mixture

The SR content in the lyophilized mixture from the OP was about four times higher than the mixture of fresh sprouts (FSP) (Table 1). The higher SR content in lyophilized sprouts prepared by the OM method is higher than in lyophilized sprouts of fresh broccoli and illustrates the increase in SR content by the addition of white radish sprouts with greater myrosinase activity. The pH of the fresh broccoli sprouts juice was 5.80, and the dry matter content was 3.66%.

The powder mixtures of broccoli and radish produced using the OP and FSP procedures had a relatively stable SR content at room temperature. The lyophilized mixture from OP had a decreased SR content after 90 days of storage at room temperature of up to 16% (from 1432 to 1210 μg/g d.w. of the mixture), and the FSP mixture of fresh sprouts decreased up to 26% (from 382 to 283 μg/g d.w. of the mixture).

### 3.2. Influence of Temperature in a Laboratory and Semi Pilot Scale

**Figure 1:**
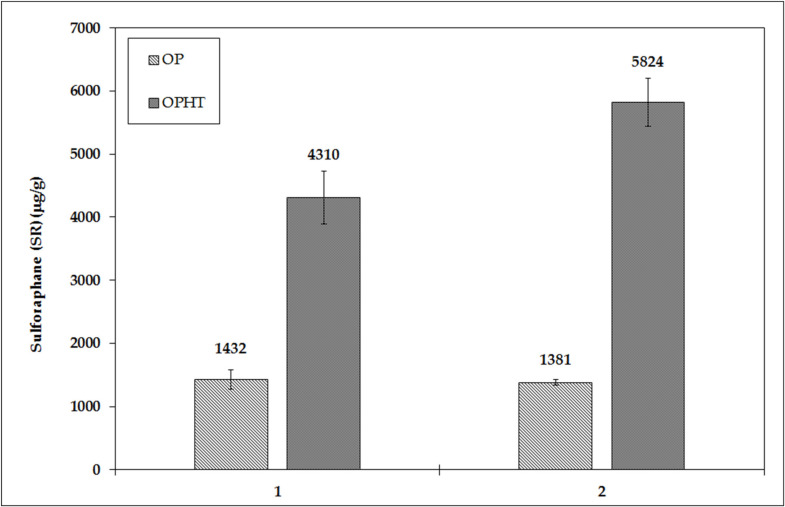
Effect of temperature on the preparation of the OP vs. OPHT lyophilized mixture on the SR content [μg/g d.w.]—Batch 1 on the left and Batch 2 on the right.

Two batches of broccoli sprouts OP powder mixture processed at 60 °C and two OPHT batches, i.e., processed at 100 °C, were prepared. The original goal of preparing the dried broccoli sprouts at a higher temperature was to inactivate both the myrosinase and ESP protein, and it was expected that the amount of SR present would be reduced at this higher temperature so that the resulting material could be used as a product with low content of SR for further studies and testing. However, it was shown that when using the OP technology at 60 °C, the SR content was around 1400 μg/g d.w. (1432 and 1381 μg/g d.w.); however, when using the OPHT technology at 100 °C, the SR content was about 3.5 times higher, about 5300 μg/g d.w. (4310 and 5824 μg/g d.w.). The higher temperature, thus, led to a significant increase in the SR content (Figure 1).

As can be seen, temperature and uniform heating of the material plays a critical role. A recently published research [11] indicated that pretreating broccoli or broccoli sprouts to 60 °C for 10 min can inactivate ESP, while retaining the myrosinase activity and, therefore, increasing the formation of SR. Thus, the prevailing opinion in the available literature is that temperatures should not exceed 60–70 °C. This is also true when using hot water to cook broccoli, although exceptions for steaming and microwaving broccoli can be found in the literature. Wang et al. [17] found that 1 min of steaming increased the yield of SR and lasted even after steaming for 3 min. Additionally, Tabart et al. [18] found that the amount of SR in broccoli increased by two-fold after steaming for 5 min and four- to six-fold after microwaving for 1 min; it is worth noting that the published data are valid for broccoli and not for broccoli sprouts.

Local overheating or underheating of the material can have a negative effect on the SR content. In addition to comparable results, the OP was done at 60 °C and the OPHT was done at 100 °C, where the temperature inside the batches was thoroughly measured. Other procedures were performed where the batch temperature was not continuously monitored MOP or due to the transfer processes with a laboratory scale (dried material of about 0.5 kg, with the addition of water up to 5.5 kg) on a semi pilot scale (dried material of about 4 or 7 kg, with the addition of water up to 45 kg). Unfortunately, the actual temperature did not correspond to the required material temperature at the point of contact with the heater during the OPHT—pilot mode. The results obtained in this way were not entirely suitable for comparison, but they point to the importance of monitoring the temperature of the batch itself.

In the modified OP designated as the MOP, where the temperature in the center of the batch did not exceed 40 °C, a significantly lower SR content of 249.09 μg/g d.w. was measured, which is approximately 5.5 times lower than the OP. 

On the semi-pilot scale indicated as the OPHT-pilot mode, there was local overheating of the powder mixture when container temperatures went above 103 °C; at the same time, part of the material evaporated when the suspension was heated with steam, and only 28.3 kg of material remained from 45 kg of starting material. Although the temperature profile was maintained at 100 °C and within the conditions of the semi-pilot plant regime, the resulting SR content was only 348.78 μg/g d.w., which was about 15 times lower than the OPHT method with values around 5000 μg/g d.w. Local overheating, together with significant evaporation of batch material, led to an enormous decrease in the SR content.

### 3.3. Influence of the Extraction Agent

**Figure 2:**
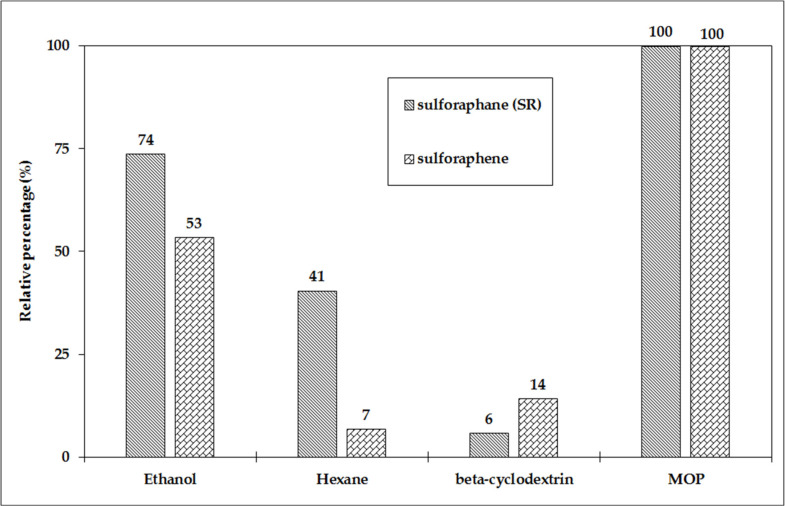
Effect of extractant on the percentage of SR and sulforaphene relative to their content in the MOP lyophilized mixture.

The broccoli and radish powder mixture produced using the MOP with an SR content of 249.09 μg/g d.w. was used as the starting material for the extraction of SR using various solvents (ethanol and hexane) and β-cyclodextrin. The extraction efficiency relative to the SR content in the original material was highest for ethanol (184 μg/g d.w.), less than half for hexane (101 μg/g d.w.), and only about 5% for β-cyclodextrin (15 μg/g d.w.) (Figure 2). In addition to SR, the sulforaphene derived from radish sprouts was also monitored. The extraction yield was higher for sulforaphene using β-cyclodextrin compared to sulforaphane.

### 3.4. Influence of Sample Preparation on Sulforaphane Analysis Using HPLC

**Figure 3:**
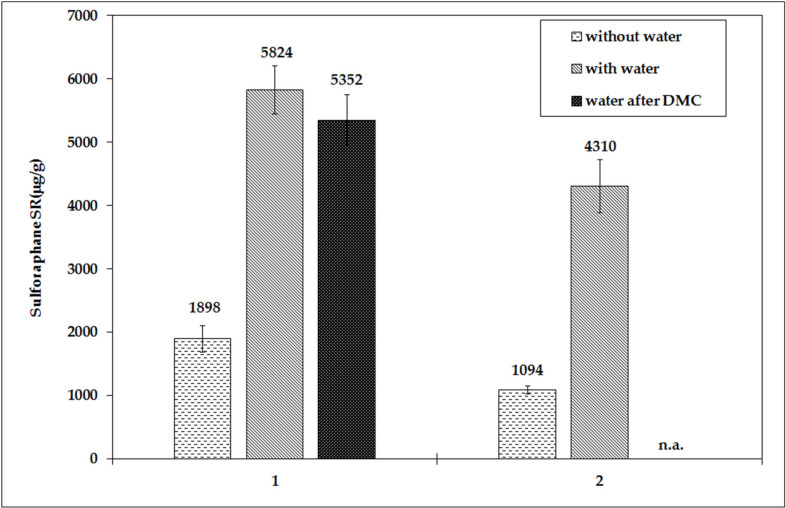
Influence of the sample preparation method on SR analysis using HPLC for lyophilized mixtures (OPHT)—Batch 1 on the left and Batch 2 on the right.

The extraction of SR into DCM was performed when preparing the sample for HPLC analysis. The determination of sulforaphane is based on the work of Bertelli et al. (1998) [19], with modification which consist of the addition of water before the addition of DCM, letting the mixture stand for 15 min, and extraction of the sample five times with DCM. In this case, a higher SR content was measured compared to the method without water addition. When using the MOP without the addition of water (M1), the values are significantly reduced (by about a third). For a powder mixture prepared at 100 °C (OPHT), the SR content was about 5000 μg/g d.w. when using water, but only about 1500 μg/g d.w. without water. When water is added immediately after the first DCM extraction step (M2), there is practically no difference in SR content, i.e., the content was around 5000 μg/g d.w. (Figure 3).

Water is, therefore, important for the conversion of GR into SR in lyophilized broccoli powder. Because the kinetics of this conversion has a certain time course, it is important to exactly follow the optimal procedures for sample preparation, especially the times, methods, and the amount of water added. In the literature, we found considerably prolonged water exposure times in samples, e.g., Li et al. [9] let the sample stand with water for 8 h in the dark and then used the salting-out effect for SR extraction. 

### 3.5. Sensory Initial Testing 

Ten volunteer assessors experienced no side effects from digestion during eating lyophilized broccoli sprouts. They observed the following effects: when added to water, part of the powder remained as a deposit at the bottom of the glass. Improper mixing or the addition of dry powder was an inhalation risk. Mixing with yogurt, buttermilk, jam, etc., seemed to be suitable ways for consuming the powder. When left open to the air, the powder absorbed moisture from the air and changed consistency. Samples from the OP process at 60 °C were beautifully green and had a typical broccoli taste, whereas samples from the OPHT process at 100 °C were brownish but still had a typical broccoli taste.

### 3.6. Microbial Quality Evaluation

**Figure 4:**
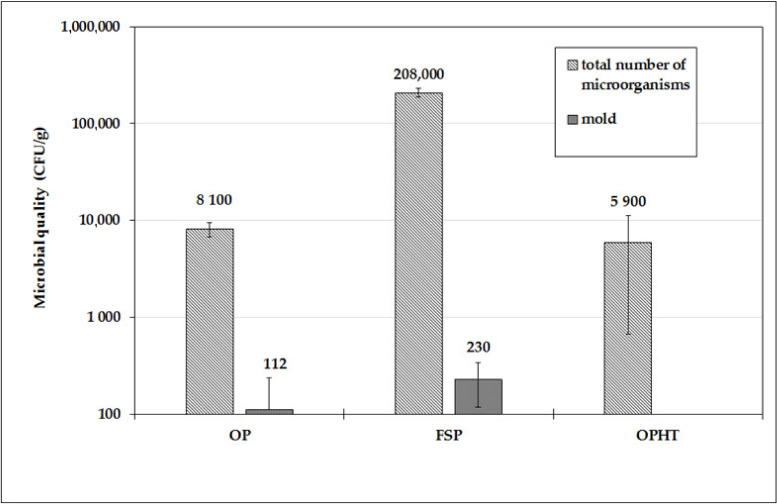
Microbial qualities in variously prepared from OP, FSP, and OPHT lyophilized mixtures.

The contents of the microorganisms were determined for the OP, MOP, and OPHT lyophilized mixtures, i.e., the total number of microorganisms (TPC), fungi, and yeast (Figure 4). The yeast content of all three types of samples was below 10^2^ CFU/g. The highest mold content was measured for fresh sprouts FSP (3.6 × 10^2^ CFU/g); for mixtures of the OP starting lyophilized sprouts, there was 3.0 × 10^2^ CFU/g, and in the OPHT sample this was below 10^2^ CFU/g. The total number of microorganisms was the highest in fresh sprouts FSP, 3.6 × 10^5^ CFU/g, while in starting mixtures OP lyophilized sprouts, it was 9.3 × 10^3^ CFU/g, and in the OPHT sample, it was 9.6 × 10^3^ CFU/g.

Samples from all technologies were safely consumable [20] both on the day of preparation and after storage for up to 90 days—values were below 10^6^. The total numbers of microorganisms were about two orders of magnitude lower for the OP and OPHT samples prepared from lyophilized sprouts than from fresh sprouts (FSP).

## 4. Conclusions

The experiments carried out show that temperature, among other parameters, plays a critical role in the transformation of GR to SR. In contrast to the generally recommended temperature of 60–70 °C at which GR is transformed, more SR was obtained at 100 °C than at 60 °C, i.e., increased temperatures increase the SR yield. However, in the event of local overheating above 100 °C, i.e., in the semi pilot plant mode (OPHT-pilot mode), this was not the case. In commercial preparation, the temperature distribution within the batch Fiche yielded a smaller amount of SR than when heated on a flat surface, i.e., the OP. 

Myrosinase and its activity is a crucial factor in the conversion of GR to SR. Under technological conditions, it is not possible or realistic to measure all theoretical parameters, e.g., the amount of GR, the myrosinase activity, of which, as described in the literature, there are several forms, and the ESP activity, especially considering that the raw material can vary. It has been shown that from a technological point of view, the most suitable approach is to ensure a constant addition of myrosinase in the form of radish (*Raphanus sativus*) sprouts, to maintain the temperatures and to determine the SR content in the final product. It seems that in the future, thermostable myrosinase from different cruciferous vegetables needs to be studied.

Emerging food technologies, such as super-heated steam blanching, ultrasonic-assisted blanching, and far-infrared blanching, should also be studied as ways of maintaining myrosinase activity.

The determination of SR using HPLC of prepared products is influenced by sample treatment during extraction with dichloromethane, in particular by the addition of water to the sample and the exposure time. Because the kinetics of GR to SR conversion has a specific time course, it is important to exactly follow the optimal procedures for sample preparation, especially the number of extractions, extraction methods and the amount of water added. 

From a sensory and microbial point of view, the production of lyophilized broccoli containing SR is not a problem. However, ensuring consistent SR content, which depends on all lyophilizate preparation conditions, can be a problem.

## Figures and Tables

**Table 1:** SR content [μg/g d.w.] in lyophilized mixtures OP and FSP and its change during storage at room temperature.

Time	0 Days	15 Days	30 Days	60 Days	90 Days
OP	1432 ± 153	1368 ± 152	1224 ± 47	1260 ± 24	1210 ± 33
FSP	382 ± 18	348 ± 25	340 ± 28	303 ± 6	283 ± 6

## Data Availability

Not applicable.
